# Methods to monitor respiratory effort during mechanical ventilation

**DOI:** 10.62675/2965-2774.20260183

**Published:** 2026-01-08

**Authors:** Glauco Marinho Plens, Bruno do Valle Pinheiro, Irene Telias, Eduardo Leite Vieira Costa

**Affiliations:** 1 Universidade de São Paulo Instituto do Coração Hospital das Clínicas São Paulo SP Brazil Respiratory Intensive Care Unit, Pulmonology Division, Instituto do Coração, Hospital das Clínicas, Faculdade de Medicina, Universidade de São Paulo - São Paulo (SP), Brazil.; 2 Universidade Federal de Juiz de Fora Hospital Universitário Pulmonary and Critical Care Division Juiz de Fora MG Brazil Pulmonary and Critical Care Division, Hospital Universitário, Universidade Federal de Juiz de Fora - Juiz de Fora (MG), Brazil.; 3 University Health Network and Sinai Health System Division of Respirology Department of Medicine Toronto Ontario Canada Division of Respirology, Department of Medicine, University Health Network and Sinai Health System - Toronto, Ontario, Canada.

Clinicians typically prioritize lung-protective targets during the initial hours of controlled mechanical ventilation in patients with acute respiratory failure. However, during the subsequent assisted phase, protective strategies tend to be less stringent, although there is growing evidence that maintaining both lung- and diaphragm-protective (LDP) targets is associated with better patient outcomes.^(1,2)^

A significant challenge in implementing LDP strategies is accurately estimating respiratory muscle effort, which, along with ventilator-delivered pressures, determines lung distension. Several physiological variables determine the magnitude of respiratory effort, including respiratory drive, neuromechanical coupling, and muscle trophism. This viewpoint focuses on methods for estimating the pressure generated by inspiratory muscles (P_mus_) during invasive mechanical ventilation, as these are directly linked to lung stress and respiratory muscle workload.

## ESOPHAGEAL PRESSURE

Esophageal manometry remains the gold standard for measuring P_mus_^(3)^ as esophageal pressure (P_es_) closely reflects pleural pressure (P_pl_) due to the esophagus's proximity to the pleural space.^(4)^ Esophageal pressure is typically measured using a balloon catheter, inserted through the nostrils or oral cavity, and connected to a pressure transducer (such as a ventilator's port or a dedicated acquisition system). Proper positioning and balloon filling are essential, as inadequate calibration can lead to inaccurate measurements of both absolute pleural pressure and its tidal swings.^(5,6)^ Esophageal and airway pressure variations during an expiratory occlusion should be approximately equal (the ratio between the change in airway and esophageal pressure should be between 0.8 and 1.2) to confirm the correct filling volume and position.^(3)^

Once the esophageal balloon is properly calibrated, P_mus_ can be calculated as the difference between the chest wall elastic recoil pressure (P_cw_) and P_es_. The P_cw_ represents the pressure needed to expand the chest wall alone. Since P_mus_ acts on the entire respiratory system, it contributes to the expansion of both the chest wall and the lungs.

Although esophageal manometry is the gold standard for assessing inspiratory muscle pressure, it requires specialized expertise for proper calibration and involves additional cost. Given these challenges, noninvasive techniques have been developed as feasible alternatives for estimating inspiratory effort at the bedside.

## AIRWAY OCCLUSION PRESSURE

Airway occlusion pressure (P_0.1_) quantifies the drop in airway pressure 100 milliseconds after the onset of inspiratory effort during an occluded breath. It provides an accurate assessment of respiratory drive, as it is unaffected by respiratory mechanics or the patient's conscious response.^(7)^ Airway occlusion pressure can be used to assess inspiratory effort, detecting low or excessive effort with reasonable accuracy. Validated cutoffs of P_0.1_ for detecting low or excessive effort per minute are < 1.0cmH_2_O and > 3.5 - 4.0cmH_2_O, respectively.^(8)^ However, in patients with high inspiratory effort, P_0.1_ may underestimate the actual effort per breath. A study in children demonstrated that the presence of high airway resistance, tidal volume, and opioid use is associated with P_0.1_ underestimation of effort.^(9)^

**Figure 1:**
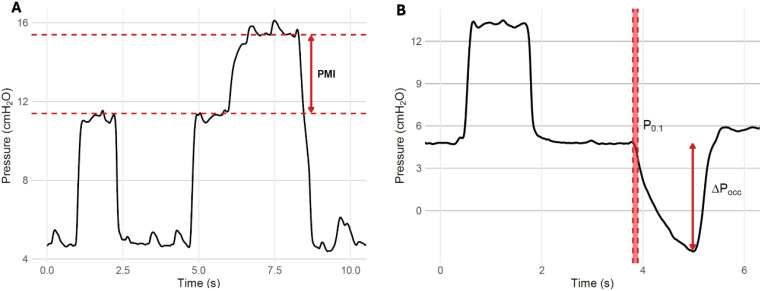
Airway pressure tracings illustrating the measurements of pressure muscle index, P_0.1_, and ΔP_occ_.

Airway occlusion pressure can be measured through an expiratory occlusion maneuver (Figure 1). Modern ventilators can also display P_0.1_ as a maneuver using a proper occlusion with direct P_0.1_ measurement or by extrapolation based on the drop in airway pressure during the trigger phase.^(8)^

(A) Pressure muscle index is calculated as the difference between plateau and peak pressures. (B) P_0.1_ is defined as the negative deflection occurring 100 milliseconds after the onset of inspiratory effort, and ΔP_occ_ represents the maximal deflection in airway pressure during an expiratory breath hold, induced by the patient's effort.

## PRESSURE MUSCLE INDEX

Pressure muscle index (PMI) is a direct estimate of P_mus_ obtained through an end-inspiratory occlusion maneuver during assisted mechanical ventilation. Unlike in controlled mechanical ventilation, in which plateau pressure is typically lower than peak airway pressure, significant inspiratory effort can result in a plateau pressure exceeding peak airway pressure. While it might seem counterintuitive, the explanation for this phenomenon is simple. The total pressure applied to the respiratory system during inflation is the sum of the pressure delivered by the ventilator and that generated by the respiratory muscles. During an end-inspiratory occlusion, relaxation of the inspiratory muscles allows the respiratory system to recoil at the inflated volume. This recoil is transmitted to the airways, resulting in a rise in airway pressure equivalent to the inspiratory muscle pressure at the onset of the occlusion. The difference between higher plateau pressure and "peak airway pressure", termed PMI, reflects the contribution of the respiratory muscles to respiratory system inflation.^(10,11)^

For reliable measurements, clinicians should ensure a horizontal plateau lasting at least 2 seconds, reached shortly after the occlusion maneuver^(12)^ (Figure 1). The PMI also allows for estimating the respiratory system static driving pressure, calculated as the plateau pressure obtained during the PMI maneuver minus PEEP.^(13)^

Notably, since the PMI does not account for the inspiratory muscle pressure exerted to overcome airway resistance, it can underestimate the total inspiratory effort, especially in patients with obstructive disorders.^(10)^ Additionally, the accuracy of respiratory system compliance and driving pressure estimates has been considered suboptimal in previous publications.^(14)^ However, in observational studies, higher driving pressure measured with an end-inspiratory occlusion during assisted ventilation was associated with increased mortality, highlighting its potential role as a target for lung protective strategies during assisted ventilation.^(2,15)^

## OCCLUSION PRESSURE

Occlusion pressure during an expiratory hold (ΔP_occ_) is another method for estimating inspiratory muscle effort. ΔP_occ_ is measured as the maximal negative deflection in the airway pressure elicited by an inspiratory effort during an occluded breath. The end-expiratory occlusion maneuver, available in most ventilators, should be long enough to capture one whole inspiratory effort exerted by the patient while ensuring there are no leaks.

P_mus_ during unoccluded assisted breaths can be estimated as 75% of ΔP_occ_ based on two independent validation cohorts.^(16,17)^ An estimation of transpulmonary driving pressure using ΔP_occ_ has also been proposed (ΔP_L,dyn_). This is calculated as the positive pressure delivered by the ventilator minus 66% of ΔP_occ._^(16,18)^ Although prospective trials indicating targets of ΔP_L,dyn_ to improve clinical outcomes are lacking, recent physiological trials have targeted ΔP_L,dyn_ ≤ 20cmH_2_O.^(19)^ Both ΔP_occ_ and ΔP_L,dyn_ have shown excellent performance in detecting high levels of P_mus_ and transpulmonary driving pressure, respectively. However, the accuracy of the absolute values for P_mus_ estimations using ΔP_occ_ may be limited, with relative errors exceeding one-third of the estimated value.^(16)^

## PRACTICAL APPROACH

At the bedside, a high respiratory drive (P_0.1_ > 3.5cmH_2_O) can be considered either appropriate or inappropriate, depending on the presence of respiratory alkalosis. Inappropriately high drive may reflect agitation, inadequate sedation or analgesia, or a response to systemic inflammation or lung injury. In such cases, increasing ventilator support is usually ineffective, may worsen alkalosis, and promote lung injury. Conversely, high drive may be appropriate in the context of increased CO_2_ production (e.g., fever, overfeeding), ventilatory inefficiency, or underassistance combined with excessive respiratory load. A high drive with low effort (P_0.1_ > 3.5cmH_2_O, P_mus_ < 5cmH_2_O) suggests respiratory muscle weakness, while low drive and effort (P_0.1_ < 1.0cmH_2_O, P_mus_ < 5cmH_2_O) indicate over-assistance or oversedation. A dynamic transpulmonary pressure (ΔP_L,dyn_) ≤ 20cmH_2_O may be a marker of lung-protective ventilation, though this threshold awaits validation.

## CONCLUSION

Monitoring respiratory effort is a crucial aspect of lung- and diaphragm-protective mechanical ventilation, particularly as patients transition from controlled to assisted ventilation. While esophageal manometry remains the gold standard for measuring inspiratory muscle pressure, it has largely been replaced by alternative methods due to its technical complexity and cost. Noninvasive techniques, such as P_0.1_, pressure muscle index, and ΔP_occ_, provide valuable insights into respiratory effort and lung stress at the bedside.

## Data Availability

The contents underlying the research text are included in the manuscript.
